# Mechanical Circulatory Support in the Very Elderly Undergoing Complex High-Risk Indicated Procedures: A Case Report and Literature Review

**DOI:** 10.3390/jcdd13030145

**Published:** 2026-03-20

**Authors:** Giuseppe Giacchi, Antonino Nicosia

**Affiliations:** Cardiology Department, Cardio-Neuro-Vascular Institute, Giovanni Paolo II Hospital, ASP 7 Ragusa, 97100 Ragusa, Italy; antonino.nicosia@asp.rg.it

**Keywords:** very elderly patient, percutaneous coronary intervention, PCI, mechanical circulatory support, MCS, complex high-risk indicated procedure, CHIP, calcified coronary lesion, coronary artery disease, CAD

## Abstract

Interventional treatment of very elderly patients with severe coronary artery disease is currently one of the central topics in interventional cardiology. Technological progress and increased life expectancy have made these patients appropriate candidates for contemporary standards of care, especially those with an active lifestyle. We hereby report the case of a 95-year-old patient hospitalized for acute myocardial infarction, who underwent a complex percutaneous coronary intervention with mechanical circulatory support. A literature review on mechanical circulatory support devices in older adults is also provided.

## 1. Introduction

Mechanical circulatory support (MCS) devices have a crucial role in interventional cardiology. They are invaluable in complex high-risk indicated procedures (CHIP) and have become a keystone in the management of acute myocardial infarction (AMI)-related cardiogenic shock [1].

An increasing life expectancy and technological advances have led to a progressive rise in the average age of patients treated in the catheterization laboratories. Patients undergoing interventional procedures are becoming progressively older and, especially those with a very active lifestyle and no prognostically limiting clinical comorbidities, deserve access to the best available therapies [2].

In this manuscript, we present the case of a very elderly patient hospitalized for an AMI and treated with MCS-assisted percutaneous coronary intervention (PCI). A brief literature review on the use of MCS in very elderly patients is also provided.

## 2. Case Report

**Figure 1:**
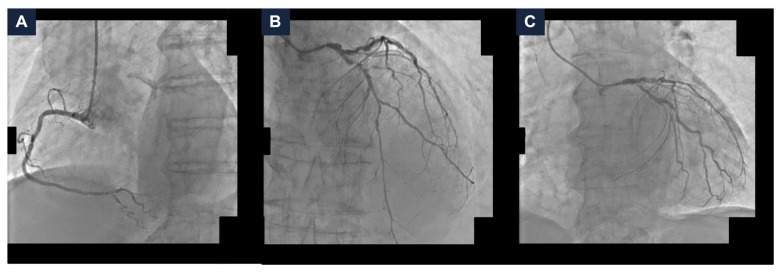
**Baseline coronary angiography.** (**A**) Right coronary artery angiography demonstrating no critical stenoses. (**B**,**C**) Left coronary artery angiography in cranial (panel **B**) and caudal (panel **C**) views showing severe, heavily calcified, sub-occlusive and critical stenoses of the left main coronary artery, proximal and mid left anterior descending artery, and ostial and proximal left circumflex artery.

A 95-year-old man with hypertension and hyperlipidemia was admitted to our hospital with Non-ST-segment elevation myocardial infarction (NSTEMI). His medical history was positive for chronic obstructive pulmonary disease and stage IV chronic kidney disease (the estimated glomerular filtration rate (eGFR) at hospitalization was 28 mL/min/1.73 m^2^). No other relevant comorbidities were reported, and he had very good cognitive and geriatric conditions. Transthoracic echocardiography was performed and revealed a moderately hypertrophic left ventricle, akinesis of the apex and anterior interventricular septum, hypokinesis of the anterior and anterolateral walls, and a left ventricular ejection fraction (LVEF) of 33%; the right ventricle had normal dimensions with a tricuspid annular plane systolic excursion of 19 mm; the mitral and tricuspid valves had moderate regurgitation, and the estimated systolic pulmonary artery pressure was 40 mmHg. Coronary angiography was scheduled. It showed a dominant right coronary artery, with a good development and caliber, and no critical stenoses. The left coronary artery had a good caliber and development, too. It presented a heavily calcified, critical stenosis of the ostial left main coronary artery (LMCA), along with multiple, severely calcified, sub-occlusive stenoses of the ostial left circumflex artery (LCx) and the proximal left anterior descending artery (LAD), and several, highly calcified, critical stenoses of the mid LAD and proximal LCx (Figure 1, panel A–C and Video S1). After the coronary angiography, a comprehensive clinical and technical evaluation of the patient’s conditions was performed, in order to determine the best therapeutic and prognostic strategy. The most relevant clinical and technical elements were as follows: the patient’s very active lifestyle, with no relevant clinical comorbidities that could have given him an unfavorable prognosis; the multiple, heavily calcified, sub-occlusive and critical stenoses of the LMCA-LAD-LCx bifurcation, that had a strong prognostic impact; and the severely depressed systolic function of the left ventricle, which conferred to the revascularization an even higher risk. MCS-assisted PCI of the LMCA-LAD-LCx bifurcation was proposed, in order to offer a prognostic benefit. The patient initially refused. Therefore, the hospitalization was extended in order to optimize the medical therapy. Antihypertensive agents (Amlodipine, Bisoprolol, and Doxazosin) and diuretics (Furosemide and Canrenone) were titrated while renal function and serum potassium levels were monitored to ensure safety and efficacy. Thus, the patient was discharged on a dual antiplatelet therapy with Ticagrelor 180 mg/day and Acetylsalicylic Acid 100 mg/day, Amlodipine 10 mg/day, Bisoprolol 5 mg/day, Furosemide 50 mg/day, Canrenone 50 mg/day, Doxazosin 8 mg/day, and a dual hypolipidemic therapy with Atorvastatin 20 mg/day and Ezetimibe 10 mg/day (at hospital discharge, the eGFR was 25 mL/min/1.73 m^2^). Two weeks later, due to persistent refractory angina at rest, the patient agreed to proceed with the scheduled intervention and was readmitted. At the time of the second hospitalization, the eGFR was 16 mL/min/1.73 m^2^; consequently, Canrenone was suspended.

After careful procedural planning, the PCI strategy was as follows: delivering an Impella CP^®^ catheter (Abiomed, Inc., Danvers, MA, USA) with echo-guided left femoral artery access (SHiP technique); performing the lesion preparation of the LMCA-LAD-LCx bifurcation using a dedicated calcium-debulking device; and using a double stent technique (T-stenting). Given that the proximal LAD and the ostial LCx lesions were sub-occlusive and probably uncrossable, we decided to perform lesion debulking using the rotational atherectomy with a 1.25 mm RotaPro burr (Boston Scientific Corporation, Marlborough, MA, USA). Although the burr was slightly undersized relative to the vessel caliber, thus increasing the risk of burr entrapment, according to our opinion, other dedicated devices or larger burrs carried an excessive risk of technical failure (inability to cross the lesions) and the no-reflow phenomenon (in the case of prolongated unsuccessful rotational atherectomy). In the event of burr entrapment, management would have involved Ping-Pong and Mother-in-Child techniques. Surgical treatment was considered the last option, due to the patient’s multiple clinical comorbidities [3,4,5].

**Figure 2:**
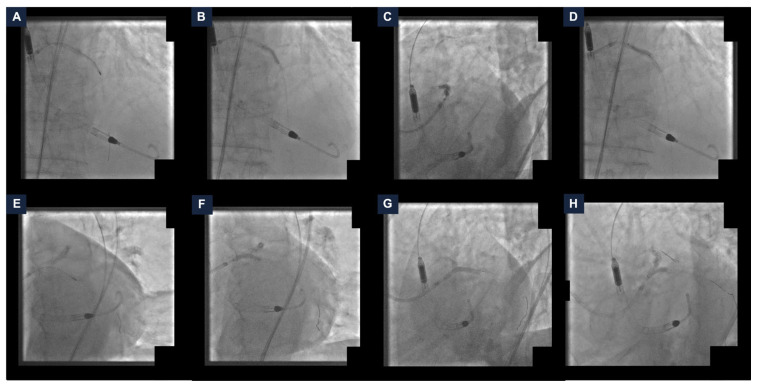
**Steps of the percutaneous coronary intervention.** (**A**) Rotational atherectomy of the left anterior descending artery (LAD). (**B**) LAD pre-dilatation with a non-compliant balloon (NC). (**C**) Drug-eluting stent (DES) deployment. (**D**) DES post-dilatation with a NC. (**E**) Rotational atherectomy of the left main coronary artery (LMCA) and the left circumflex artery (LCx). (**F**) LMCA-LCx pre-dilatation with a NC. (**G**) DES implantation. (**H**) Kissing-balloon technique on the LMCA-LAD-LCx using two NC. DES: drug-eluting stent; LAD: left anterior descending artery; LCx: left circumflex artery; LMCA: left main coronary artery; NC: non-compliant balloon.

The left common femoral artery was cannulated under ultrasound guidance. Two PercloseTM ProStyleTM devices (Abbott Vascular, Santa Clara, CA, USA) were rotated and pre-implanted at the ten and two o’clock position (“Perclose” method), and the Impella CP^®^ catheter was delivered with no complications. An EBU LauncherTM 7 Fr Guide Catheter (Medtronic, Galway, Ireland) was inserted through the Impella CP^®^ sheath using the SHiP technique. The coronary interventions itself started with calcium lesion debulking. Thus, the rotational atherectomy of the proximal LAD was performed with a 1.25 mm burr. The LAD lesion preparation was completed using a 2.0 mm semi-compliant balloon and a 3.0 non-compliant balloon (NC). A 3.0/33 mm drug-eluting stent (DES) was deployed and adequately post-dilated with a 3.0 mm NC (Figure 2, panel A–D). Subsequently, the rotational atherectomy of LMCA-LCx was performed with a 1.25 mm burr. Pre-dilatation with a 3.0 mm NC was carried out and a 3.50/34 mm DES was implanted. MCS-assisted PCI was completed with the kissing-balloon technique on the LMCA-LAD-LCx bifurcation with two 3.0 mm NC (Figure 2, panel E–H).

**Figure 3:**
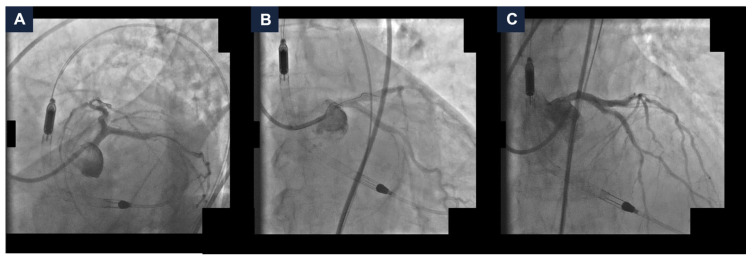
**Final complex high-risk indicated procedure result.** Final coronary angiography demonstrating a good final result with Thrombolysis In Myocardial Infarction grade 3 flow in spider (panel **A**), caudal (panel **B**), and cranial (panel **C**) views.

The final angiographic result was satisfactory, with a Thrombolysis in Myocardial Infarction (TIMI) grade 3 flow (Figure 3 and Video S2). The hemodynamics remained stable, so the MCS device was removed at the end of the procedure, with no vascular access complications. A total of 40 mL of Iodixanol 270 mg I/mL was administered. Post-procedurally, the eGFR reached 13 mL/min/1.73 m^2^. The patient was managed with intravenous hydration. The serum potassium did not exceed 5.0 mEq/L and the urine output remained stable. After a few days, the kidney function returned to baseline values (eGFR 27 mL/min/1.73 m^2^). No in-hospital adverse events occurred. The patient remained free from angina at the one-year follow-up [6].

## 3. Discussion

Interventional treatment of older adults with AMI and/or cardiogenic shock is an important issue in interventional cardiology. It represents one of the most important clinical matters in percutaneous coronary revascularization and has been evaluated in several papers.

For the purpose of this review, we conducted a comprehensive PubMed search, and selected all published studies reporting the clinical outcomes of elderly subjects (≥70 years old) treated with transcatheter MCS. We categorized these studies into single-arm registries and two-arm studies (Table 1).

### 3.1. Single-Arm Registries

Seguchi M. et al., in their retrospective, single-center registry, investigated the clinical outcomes in elderly patients (≥80-year-old) hospitalized for AMI and treated with culprit lesion PCI. The primary clinical endpoint was in-hospital mortality. The pre-admission activity of daily living was evaluated with the modified KATZ index, a mathematical tool used to assess the geriatric status in elderly subjects. The primary clinical outcome rate was 10.2% (*n* = 42). In multivariate logistic regression analysis, cardiac arrest (odds ratio (OR) 4.642, 95% confidence interval (CI) 1.177–18.305, *p* = 0.028), Killip class IV versus class I (OR 5.732, 95% CI 1.076–16.630, *p* = 0.001), modified KATZ index (OR 1.212, 95% CI 1.001–1.469, *p* = 0.049), hemoglobin level (OR 0.803, 95% CI 0.656–0.983, *p* = 0.033), pacemaker use (OR 2.603, 95% CI 1.010–6.709, *p* = 0.048), final TIMI flow grade 3 versus ≤2 (OR 0.240, 95% CI 0.093–0.618, *p* = 0.003), and MCS use (OR 4.264, 95% CI 1.818–10.005, *p* = 0.001) were associated with in-hospital mortality [7].

Damluji A.A. et al., in their retrospective analysis, evaluated the clinical outcomes in older adults (≥75 years) hospitalized in the United States of America for ST-segment elevation myocardial infarction (STEMI) complicated by cardiogenic shock between 1999 and 2013. The first clinical endpoint evaluated was in-hospital mortality.

The proportion of older adults presenting with STEMI and shock declined over the years analyzed (proportion of older adults with STEMI and shock: 1999: 42%, 2013: 29%). The rate of percutaneous revascularization in older adults increased over the time (27% vs. 56%, *p* < 0.001), while the in-hospital mortality decreased (64% vs. 46%, *p* < 0.001). By applying propensity-score-matching methods, percutaneous revascularization was associated with a lower risk of in-hospital mortality (Mantel–Haenszel OR 0.48, 95% CI 0.45–0.51) [8].

**Table 1:** Published studies.

Study Title	Study Type/Design	Number of Patients	Elderly Patients (%)	Primary Outcome	Reference Number
Alonso-Fernandez-Gatta M. et al.	Retrospective, single-center study, comparing consecutive elderly patients (≥70 years old) with hemodynamic instability treated by Impella CP^®^ or VA-ECMO vs. younger patients (<70 years)	164	27.4	Weaning failure (death during hemodynamic support, within 24 h after MCS removal, or death due to cardiogenic shock, heart failure, or cardiac arrest during hospitalization): elderly 40% vs. younger 43.7%, *p* = 0.403	[9]
Damluji A.A. et al.	Retrospective, multi-center registry, enrolling consecutive older adults (≥75 years old) hospitalized for STEMI complicated by cardiogenic shock between 1999 and 2013	111,901	100	In-hospital mortality: 1999 64% vs. 2013 46%; *p* < 0.001	[8]
JAMIR	Retrospective, multi-center study, comparing consecutive Japanese patients with STEMI complicated by cardiogenic shock and treated by PCI vs. patients treated with optimal medical therapy	2760	28.6	All-cause mortality: PCI 31.3% vs. medical therapy 56.0%, *p* < 0.001 Cardiac death: PCI 24.2% vs. medical therapy 45.9%, *p* < 0.001 Sub-analysis elderly patients (≥80 years old) vs. younger patients (<80 years): All-cause mortality: elderly 45.8% vs. younger 30.1%, *p* < 0.001 Cardiac death: elderly 38.3% vs. younger 22.5%, *p* < 0.001	[10]
Seguchi M. et al.	Retrospective, single-center registry, enrolling consecutive elderly patients (≥80-year-old) hospitalized for AMI and treated with culprit-lesion PCI	412	100	In-hospital mortality = 10.2%	[7]
Vallabhajosyula S. et al.	Retrospective, multi-center study, comparing consecutive elderly (≥75 years old) male patients hospitalized for AMI complicated by cardiogenic shock vs. elderly (≥75 years old) female patients	134,501	100	In-hospital mortality: female 56.6% vs. male 55.1%, unadjusted OR 1.06, 95% CI 1.04–1.09, *p* < 0.001	[11]

AMI: acute myocardial infarction; CI: confidence interval; MCS: mechanical circulatory support; OR: odds ratio; PCI: percutaneous coronary intervention; STEMI: ST-segment elevation myocardial infarction; VA-ECMO: veno-arterial extracorporeal membrane oxygenation.

### 3.2. Two-Arm Studies

Alonso-Fernandez-Gatta M. et al., in their retrospective registry, compared the clinical outcomes between elderly (≥70 years old) and young (<70 years old) patients with a hemodynamic instability treated by Impella CP^®^ or veno-arterial extracorporeal membrane oxygenation (VA-ECMO) implantation. The primary endpoint was the weaning failure, defined as death during hemodynamic support, within 24 h after MCS removal, or death due to cardiogenic shock, heart failure, or cardiac arrest during hospitalization. No significant differences in survival were observed between groups, both at discharge (elderly 40% vs. young 43.7%, *p* = 0.403) and at the median follow-up of 13.6 months (35.6% vs. 37.8%, *p* = 0.469). Elderly patients treated with MCS for CHIP support had a higher survival rate compared to those treated for bridge indications (*p* = 0.013). At multivariate analysis, the lactate level at implantation was the only independent predictor of survival in the elderly group (hazard ratio (HR) 0.742, 95% CI 0.592–0.931, *p* = 0.010) [9].

The JAMIR is a retrospective, multi-center registry. It evaluated the clinical outcomes in Japanese patients hospitalized for STEMI complicated by cardiogenic shock and treated by PCI or optimal medical therapy. The primary clinical endpoints were all-cause in-hospital mortality and in-hospital cardiac death. Both the all-cause in-hospital mortality and in-hospital cardiac death rates were lower in the PCI arm (all-cause mortality: PCI group 31.3% vs. medical therapy group 56.0%, *p* < 0.001; cardiac death: 24.2% vs. 45.9%, *p* < 0.001). In multivariate logistic regression, PCI was a predictor of lower mortality rates (all-cause mortality: OR 0.508, 95% CI 0.347–0.744, *p* < 0.001; cardiac death: OR 0.493, 95% CI 0.335–0.729, *p* < 0.001). The JAMIR investigators also compared the clinical outcomes between patients aged ≥ 80 years (*n* = 789—28.6%) and those aged < 80 years (*n* = 1971—71.4%). Both clinical outcomes were more frequent in elderly patients (all-cause mortality: ≥80-year arm 45.8% vs. <80-year arm 30.1%, *p* < 0.001; cardiac death: 38.3% vs. 22.5%, *p* < 0.001). In the <80 years old group, PCI was a predictor of both primary clinical endpoints (all-cause mortality: OR 0.461, 95% CI 0.283–0.754; cardiac death: OR 0.470, 95% CI 0.284–0.788). Conversely, in elderly patients, PCI was independently associated with a lower in-hospital cardiac death rate (adjusted OR 0.524, 95% CI 0.281–0.975), but not with the all-cause in-hospital mortality (adjusted OR 0.564, 95% CI 0.300–1.050) [10].

Vallabhajosyula S. et al., in their retrospective, multi-center registry, compared clinical outcomes between older male and female patients (≥75 years) admitted for AMI complicated by cardiogenic shock. The primary clinical endpoint was in-hospital mortality stratified by sex. The female subjects had higher in-hospital mortality (56.6% vs. 55.1%—*p* < 0.001). On a multivariable logistic regression, the female sex was independently associated with increased in-hospital mortality (OR 1.05, 95% CI 1.02–1.08, *p* < 0.001) [11].

The treatment of older adults with AMI and/or cardiogenic shock is challenging. This is due to the high prevalence of comorbidities, that are more frequent with an advancing age, the frailty of these patients—which often impacts compliance and the efficacy of the medical treatments, the presence of more severe and more complex coronary artery disease (CAD), and the more frequent presence of severe left ventricle dysfunction compared to younger adults. However, as a result of the improvements in medical care, these patients sometimes have good clinical conditions and very active lifestyles, with a biological age that is often lower than the chronological one. Consequently, transcatheter interventions have become a valuable option in these patients, when the general conditions and neurological status are preserved.

In the literature, the data on the treatment of very elderly subjects with AMI and/or cardiogenic shock are somewhat inconsistent. Although the data suggest that this specific population could benefit from the current standard-of-care invasive strategies, the evidence is too weak, due to the limited number of elderly patients in published studies and the difficulty of conducting dedicated trials for this clinically heterogeneous cohort.

The data about PCI in elderly patients are limited. In the SENIOR-RITA Trial, no survival benefit was observed with PCI versus the optimal medical therapy in patients hospitalized for NSTEMI at long-term follow-up. Notably, severe, multiple comorbidities and frailty were not exclusion criteria. Moreover, Kohansal E. et al., in their meta-analysis, demonstrated no differences in the all-cause and cardiovascular mortality rates between invasive strategies and conservative management. In subjects with CAD and a reduced LVEF, no mortality difference has been demonstrated between PCI and CABG. Conversely, the use of MCS during CHIP has been associated with improved long-term outcomes compared to unsupported PCI. As the technological improvement and newly available devices have led to improved clinical outcomes for high-risk patients, older adults are now candidates for mechanical hemodynamic support in CHIP and cardiogenic shock. Taking into account also the increasing life expectancy, advanced age alone should not preclude subjects from being candidates for hemodynamic support. However, a holistic evaluation of elderly patients is mandatory, in order to properly select those who could benefit most from MCS. Geriatric conditions play a prominent role in the peri-procedural PCI risk in this subset of patients: they have a high prevalence and a strong association with clinical outcomes. Therefore, an optimal and standardized risk stratification is of crucial importance. It should integrate four pillars: the cardiovascular risk (assessed by the risk scores), the hemodynamic risk (determined by the cardiac conditions), the anatomic and procedural risk (that depends on the anatomical and procedural aspects), and the geriatric syndromes (delirium, dementia, disability, falls, frailty, incontinence, multimorbidity, polypharmacy, and sarcopenia). The cardiovascular risk assessment includes several data points regarding the patient’s demographics and comorbidities, the clinical presentation, the physical examination, the diagnostic tools, and the several cardiological risk scores used to predict prognosis, and thrombotic and bleeding risk (TIMI, GRACE, GRACE 2.0, and CRUSADE). Another milestone in risk stratification for patients who are candidates for percutaneous revascularization is the patient’s hemodynamic status. The conditions with the greatest impact on the prognosis are cardiac arrest at presentation, decompensated heart failure with a reduced ejection fraction, recurrent and/or persistent resting angina with electrical or hemodynamic instability, hemodynamic instability secondary to mechanical complications, and valvular heart disease. The anatomic and procedural risk is based on the presence of several high-risk anatomical features, such as left main disease, proximal LAD disease, multivessel disease, the presence of any coronary chronic total occlusions, severe calcifications requiring the use of dedicated devices, a long lesion length (>60 mm), and in-stent thrombosis. Among the anatomic and procedural aspects, the necessity of calcium-dedicated devices, the need for mechanical hemodynamic support, and bleeding preventing strategies are of paramount importance in elderly subjects. The geriatric syndromes are the fourth domain of risk assessment in older adults. These are clinical conditions with multiple risk factors, and can be divided into several main groups, according to the most affected area. The physical function domain incorporates the syndromes that affect the patient’s ability to complete the activities of daily life. It includes frailty, sarcopenia, disability, and falls. The mind and emotional domain involves conditions that impairs cognition and emotions. These are cognitive impairment, delirium, and depression. The medical domain includes disorders related to chronic conditions, such as multimorbidity, polypharmacy, and incontinence. Therefore, the clinical decision-making process must be individualized, taking into account the specific patient treatment goals and the expected potential clinical benefits. Futile or excessively harmful procedures should be avoided whenever possible [12,13,14,15,16,17].

The present report describes a clinical case of a very elderly patient hospitalized for myocardial infarction. The patient had a very active lifestyle, good geriatric conditions, and no other relevant comorbidities that could have affected his prognosis. He had a severely depressed left ventricular systolic function and heavily calcified, sub-occlusive and critical stenoses of the LMCA, LAD, and LCx. After having performed in the Heart Team a comprehensive evaluation of the clinical, technical, and geriatric factors, the MCS-assisted revascularization of the LMCA-LAD-LCx bifurcation was proposed. This invasive therapeutic strategy was safe and effective, and the patient had no clinical adverse events at the one-year follow-up.

Based on our experience, MCS is a valuable therapeutic option in older adults with AMI-related cardiogenic shock and/or those undergoing CHIP. However, not all very elderly patients are suitable candidates for advanced interventional strategies. Older adults frequently present with several comorbidities and more complex CAD. Therefore, interventional procedures have a higher procedural risk in these patients, and this must be taken into account in the pre-procedural evaluation, as the risk of futile or excessively risky interventions, offering no real clinical benefit to the treated patients, is extremely high and is often underestimated. Clinical judgment is critical, as very elderly subjects should not be excluded from invasive interventions and complex revascularization procedures based solely on age in the absence of other relevant clinical contraindications. Therefore, a holistic patient evaluation performed by a multidisciplinary Heart Team, including a geriatric specialist, should be mandatory, in order to offer the best patient-centered care. The role of MCS in very elderly patients warrants further evaluation in large studies with a long-term follow-up.

Our manuscript presents several limitations that must be taken into account. First, it is not a clinical study, but the description of a single case of an older subject undergoing a complex percutaneous revascularization for myocardial infarction. The patient had a good life status, an active lifestyle, and excellent geriatric conditions. After a comprehensive clinical evaluation, we decided to treat him with hemodynamic support device-assisted percutaneous revascularization. A comprehensive review of the clinical data published on MCS use in elderly subjects is also provided. Second, very elderly patients still represent a limited proportion of subjects treated in catheterization laboratories. Even if, with increasing life expectancy and improved medical care, the number of older adults with good clinical conditions has grown in the last few years, they are still a limited part of subjects treated in daily practice. Third, very elderly patients are underrepresented in the published literature. They are usually a minority of the subjects enrolled, or are frequently excluded from observational registries and randomized controlled trials. Therefore, the clinical evidence regarding interventional treatment in this clinical scenario is very weak and precludes the derivation of definitive clinical indications. Last, but not least, published studies on the use of MCS in older adult patients are few. Data on the indications and therapeutic options in elderly subjects remain limited. This specific subset of patients includes an heterogenous population in which the comorbidities, the geriatric syndromes, and the cardiologic conditions can vary substantially between the individuals. Consequently, it is challenging to thoroughly investigate the clinical outcomes and draw firm conclusions with adequate statistical power in this specific patient population. Therefore, the current clinical evidence remains limited.

## 4. Conclusions

Interventional treatment of older adults with AMI and/or cardiogenic shock is one of the most debated topics in interventional cardiology, given the increasing life expectancy and the technological improvements that have led to improved clinical outcomes even in very elderly patients.

This manuscript reports an MCS-assisted CHIP in a 95-year-old subject hospitalized for AMI. The patient had excellent clinical conditions and a good life expectancy. Thus, an advanced interventional strategy was proposed. This was safe and effective, and the patient had no clinical events at the one-year follow-up.

Larger series with a longer follow-up are warranted to definitely assess this clinical issue.

## Data Availability

The original contributions presented in this study are included in the article/Supplementary Materials. Further inquiries can be directed to the corresponding author.

## Supplementary Materials

The following supporting information can be downloaded at: https://www.mdpi.com/article/10.3390/jcdd13030145/s1, Video S1—Diagnostic coronary angiography: baseline coronary angiography showing a dominant right coronary artery with no critical stenoses. The left coronary artery angiography demonstrated severe, heavily calcified, sub-occlusive and critical stenoses of the left main coronary artery, the ostial and the proximal left circumflex artery, and the proximal and mid left anterior descending artery. Video S2—Final angiographic result: final mechanical circulatory support-assisted procedure result was good with Thrombolysis in Myocardial Infarction grade 3 flow; no complications occurred.

## Abbreviations

The following abbreviations are used in this manuscript:

AMI	Acute myocardial infarction
CAD	Coronary artery disease
CHIP	Complex high-risk indicated procedures
CI	Confidence interval
CABG	Coronary artery bypass grafting
DES	Drug eluting stent
eGFR	Estimated glomerular filtration rate
HR	Hazard ratio
LAD	Left anterior descending artery
LCx	Left circumflex artery
LMCA	Left main coronary artery
LVEF	Left ventricle ejection fraction
MCS	Mechanical circulatory support
NC	Non-compliant balloon
NSTEMI	Non-ST-segment elevation myocardial infarction
OR	Odds ratio
PCI	Percutaneous coronary intervention
RR	Risk ratio
STEMI	ST-segment elevation myocardial infarction
TIMI	Thrombolysis in Myocardial Infarction
VA-ECMO	Veno-arterial extracorporeal membrane oxygenation
